# Genetic drivers of metastatic dissemination in sonic hedgehog medulloblastoma

**DOI:** 10.1186/s40478-014-0085-y

**Published:** 2014-07-25

**Authors:** Noah C Jenkins, Ricky R Kalra, Adrian Dubuc, Walavan Sivakumar, Carolyn A Pedone, Xiaochong Wu, Michael D Taylor, Daniel W Fults

**Affiliations:** Department of Neurosurgery, Clinical Neurosciences Center, University of Utah School of Medicine, 175 North Medical Drive East, Salt Lake City, UT 84132 USA; Department of Pathology, Brigham and Women’s Hospital, Boston, Massachusetts USA; Division of Neurosurgery, Arthur and Sonia Labatt Brain Tumour Research Center, and Program in Developmental and Stem Cell Biology, The Hospital for Sick Children, University of Toronto, Toronto, Ontario Canada

**Keywords:** Medulloblastoma, Metastasis, Leptomeningeal dissemination, Arnt, Gdi2

## Abstract

Leptomeningeal dissemination (LMD), the metastatic spread of tumor cells via the cerebrospinal fluid to the brain and spinal cord, is an ominous prognostic sign for patients with the pediatric brain tumor medulloblastoma. The need to reduce the risk of LMD has driven the development of aggressive treatment regimens, which cause disabling neurotoxic side effects in long-term survivors. Transposon-mediated mutagenesis studies in mice have revealed numerous candidate metastasis genes. Understanding how these genes drive LMD will require functional assessment using in vivo and cell culture models of medulloblastoma. We analyzed two genes that were sites of frequent transposon insertion and highly expressed in human medulloblastomas: *Arnt* (aryl hydrocarbon receptor nuclear translocator) and *Gdi2* (GDP dissociation inhibitor 2). Here we show that ectopic expression of *Arnt* and *Gdi2* promoted LMD in mice bearing Sonic hedgehog (Shh)-induced medulloblastomas. We overexpressed *Arnt* and *Gdi2* in a human medulloblastoma cell line (DAOY) and an immortalized, nontransformed cell line derived from mouse granule neuron precursors (SHH-NPD) and quantified migration, invasiveness, and anchorage-independent growth, cell traits that are associated with metastatic competence in carcinomas. In SHH-NPD cells. *Arnt* and *Gdi2* stimulated all three traits. In DAOY cells, *Arnt* had the same effects, but *Gdi2* stimulated invasiveness only. These results support a mechanism whereby *Arnt* and *Gdi2* cause cells to detach from the primary tumor mass by increasing cell motility and invasiveness. By conferring to tumor cells the ability to proliferate without surface attachment, *Arnt* and *Gdi2* favor the formation of stable colonies of cells capable of seeding the leptomeninges.

**Electronic supplementary material:**

The online version of this article (doi:10.1186/s40478-014-0085-y) contains supplementary material, which is available to authorized users.

## Introduction

An important goal in designing therapies for patients with the malignant brain tumor medulloblastoma is to reduce the risk of metastasis. A defining characteristic of metastasis in medulloblastoma is leptomeningeal dissemination (LMD), the spread of tumor cells via the cerebrospinal fluid (CSF) to the leptomeningeal spaces of the brain and spinal cord. The need to minimize metastasis risk is critical because survival times are very low once LMD has occurred. Guided by this principle, pediatric oncologists have designed multimodality treatments, which combine surgery, chemotherapy, and craniospinal radiation [1,2]. These aggressive regimens reduce the risk of metastasis, but they are associated with disabling side effects, including neuropsychiatric challenges, stunted body growth, hormonal imbalance, epilepsy, and stroke in long-term survivors [3-5].

Because the prospect for long-term survival is so poor for patients with LMD, radiation to the entire neuraxis remains an indispensable part of medulloblastoma treatment. Unfortunately, intellectual capacity and academic achievement decline even in children treated with protocols that use reduced radiation doses [6]. Hope for prolonging disease-free survival and eliminating treatment-related neurotoxicity rests on developing therapies that specifically target the molecular mediators of LMD, our knowledge of which is limited.

Analysis of the medulloblastoma transcriptome in large cohorts of patients has shown that medulloblastomas do not comprise a uniform disease entity, but rather a diverse set of tumors, which have different gene expression profiles, rates of metastatic dissemination, and patient survival times [7-9]. Despite the identification of molecular signatures with prognostic implications, metastasis stage remains a critical determinant of high-risk tumors [10]. The expression signature of most common medulloblastoma subtype affecting infants and adults implicates the Sonic Hedgehog (Shh) signaling pathway in tumor pathogenesis. Shh signaling, which governs many aspects of embryogenesis, stimulates proliferation and inhibits differentiation of neural progenitor cells in the cerebellum [11]. Consistent with this developmental function, genetically engineered mouse models have shown that aberrant activation of the Shh pathway in either multipotent neural stem cells or granule neuron precursor cells (GNPs) in the developing cerebellum can initiate medulloblastoma formation [12-17].

We reported previously that ectopic expression of genes encoding Eras (embryonic stem cell–expressed Ras), Lhx1 (LIM-class homeobox gene 1), Ccrk (cell cycle–related kinase), and the phosphatidyl inositol 3-kinase signaling protein Akt shifted the growth characteristics of Shh-induced medulloblastoma from a localized pattern to one in which tumor cells seeded the leptomeninges of the brain and spinal cord [18]. The idea that these proteins might be LMD drivers came from a screen for metastasis genes using transposon-mediated mutagenesis in Patched-deficient mice, which develop medulloblastomas spontaneously [19].

Extensive evidence from the field of epithelial cancer metastasis indicates that a large number of genes choreograph the multistep process whereby tumor cells breach the basement membrane in the originating tissue, intravasate into the bloodstream, and colonize distant organs [20,21]. Although the chain of events involved in LMD is different from the invasion-metastasis cascade of carcinomas, the changes in cell physiology needed for medulloblastoma cells to detach from the tumor mass, enter the CSF, and proliferate in the subarachnoid spaces no doubt require an equivalently diverse set of genes.

To expand the set of LMD-driving genes, we transferred and expressed *Arnt* (aryl hydrocarbon receptor nuclear translocator) and *Gdi2* (GDP dissociation inhibitor 2), which had been identified previously as common insertion sites for the Sleeping Beauty (SB) transposon, in cerebellar neural progenitor cells in mice by retroviral transfer in combination with Shh. Here we show that ectopic expression of *Arnt* and *Gdi2* promotes spinal LMD in mice bearing Shh-induced medulloblastomas and demonstrate the effects of these genes on the motility, invasiveness, and anchorage-independent growth of medulloblastoma tumor cells and precursor cells in culture.

## Materials and methods

### Retroviral vector construction

Construction of RCAS-Shh, which contains an in-frame, carboxy-terminal epitope tag consisting of six repeats of the influenza virus hemagglutinin (HA) epitope, was described previously [14]. The cDNA clones for mouse *Arnt* and *Gdi2* were obtained from the American Type Culture Collection (Manassas, VA), where they were deposited by the Integrated Molecular Analysis of Genomes and their Expression (IMAGE) consortium (http://www.imageconsortium.org). RCAS vectors were prepared by ligating a PCR-generated cDNA corresponding to the complete coding sequence into the parent retroviral vector RCASBP(A) [22]. RCAS-Gdi2 contained an internal ribosome entry site (IRES) coupled to the *Aequorea coerulescens* green fluorescent protein (GFP) for tracking the cellular localization of the expressed proteins. To produce live virus, we transfected plasmid versions of RCAS vectors into immortalized chicken fibroblasts (DF-1 cells) and allowed them to replicate in culture.

### In vivo somatic cell gene transfer in transgenic mice

The use of mice in this study was approved by the Institutional Animal Care and Use Committee of the University of Utah. To induce medulloblastomas in mice, we used a version of the RCAS/*tv-a* somatic cell gene transfer system to transfer and express the *Shh* gene in Nestin-expressing cells in the cerebellum. Nestin, an intermediate filament protein, is a marker for neural progenitor cells prior to neuronal or glial differentiation. The RCAS/*tv-a* system uses a replication-competent, avian leukosis virus, splice acceptor (RCAS) vector, derived from the subgroup A avian leukosis virus (ALV-A), and a transgenic mouse line (*Ntv-a*) that produces TVA (the cell surface receptor for ALV-A) under control of the *Nestin* gene promoter [23]. After TVA-mediated infection of mammalian cells with RCAS retrovirus, the newly synthesized provirus integrates into the host cell genome where the transferred gene is expressed constitutively. RCAS-transduced mammalian cells do not produce infectious virus because mRNA splicing events remove the retroviral genes necessary for viral replication.

To initiate gene transfer, we injected retrovirus packaging cells (DF-1 cells transfected with and producing recombinant RCAS retrovirus) into the lateral cerebellum of the mouse from an entry point just posterior to the lambdoid suture of the skull (bilateral injections of 10^5^ cells in 1–2 μl of phosphate buffered saline (PBS)). For experiments involving simultaneous transfer of two genes, we prepared cell pellets by mixing equal numbers of both retrovirus-producing cells. We injected mice within 72 hours after birth because the number of Nestin^+^ cells decreases progressively during the course of neuronal differentiation. The mice were sacrificed when signs of increased intracranial pressure became apparent, indicated by enlarging head circumference (a sign of hydrocephalus), head tilt, gait ataxia, or failure to eat or drink. Asymptomatic mice were sacrificed 4 months after injection. The brains were fixed in formalin, and divided into quarters by parallel incisions in the coronal plane. To identify spinal LMD, we fixed whole spinal column preparations in formalin for 48–72 hours and then removed the spinal cord by microdissection. Brain and spinal cord specimens were embedded in paraffin and sectioned for histochemical analysis.

### Immunocytochemistry and microscopy

Methods for immunoperoxidase staining of mouse brain and spinal cord sections were described previously [18]. We used the following antibodies from the indicated commercial sources: Mab9E10—c-Myc (Santa Cruz Biotechnology, Santa Cruz, CA); Mab3580—GFP (Chemicon, Temecula, CA); ab14545—βIII-tubulin (Abcam, Cambridge, MA); Mab2F11—70 kDa neurofilament protein (Dako, Carpinteria, CA). Tissue sections were visualized using a Zeiss Axiovert 200 microscope, and photomicrographs were captured using an AxioCam high-resolution CCD camera and Axiovision imaging software (Carl Zeiss International, Germany).

### Gene transfer by lentivirus

To express *Arnt* and *Gdi2* in cultured cells, we used the HIV-ZsGreen lentivirus system [24]. The gene-transfer plasmid (pHIV-ZsGreen, Addgene plasmid 18121) encodes a GFP from the reef coral *Zoanthus* to track expression of the transgene in infected cells. To produce live virus for gene transfer, we ligated *Arnt* and *Gdi2* cDNAs into the NotI-XbaI site of pHIV-ZsGreen and transferred the recombinant plasmids to HEK293T packaging cells by transfection in combination with helper plasmids pMDLgRRE, pRSV-Rev, and pCMV-VSV-G. Virus particles were collected by ultracentrifugation and applied to recipient cells in tissue culture plates under biosafety level 2 conditions. Viral titer was determined from the ratio of fluorescent to nonfluorescent cells.

### In vitro scratch assay

SHH-NPD or DAOY cells, previously infected with HIV-ZsGreen lentivirus and expressing *Arnt* or *Gdi2*, were seeded into 6-well plates (35-mm well diameter) in triplicate and incubated at 37°C under 5% CO_2_ until the cell density reached 90% confluence. The cells were washed with PBS and incubated overnight in serum-free Dulbecco’s modified Eagle medium (DMEM) containing 10 μg/ml mitomycin C to suppress proliferation. A linear wound was created in the nearly confluent cell monolayer by scratching across the center of the plate using a sterile 200-μl pipette tip in a smooth, even fashion, and the medium was replaced with DMEM containing 10% fetal bovine serum. Digital photomicrographs were taken 0 and 10 hours after making the scratch wound through a 10× phase contrast microscope objective. Prior to each photo session, the medium was replaced with PBS to obtain a clear photographic image. To quantify the rate of cell migration across the wound gap, we measured the distance between the two advancing, converging cell fronts at five points equidistant along the scratch wound. The rate of cell migration (μm/hour) was calculated using the following formula: [average scratch width at 0 hours − average scratch width at 10 hours] ÷ 20. The denominator of 20 is used to account for the ten hours of observation and two advancing cell fronts. Each experiment was repeated on a separate day. Standard deviation (SD) for the mean migration rate was derived from 30 data points.

### Matrigel chemoinvasion assay

SHH-NPD or DAOY cells (2 × 10^5^), previously infected with HIV-ZsGreen lentivirus and expressing *Arnt* or *Gdi2*, were plated into the upper chamber of an 8-μm, 6-well BD Biocoat Matrigel invasion chamber (BD Biosciences, Bradford, MA) in 500 μl of serum-free DMEM. Prior to plating, the cells were treated with 10 μg/ml mitomycin C to suppress proliferation. Each bottom chamber was filled with 2 ml of DMEM containing 10% fetal bovine serum and then incubated for 24 hours at 37°C under 10% CO_2_. After incubation, the noninvading cells are removed from the upper chamber with a cotton swab. The invading cells on the lower surface of the membrane were stained with Hema 3 (Fisher Scientific, Loughborough, UK) and counted under a microscope. Five nonoverlapping microscope fields (10× objective) were counted in each well. Each assay was repeated, and the mean number of invading cells per well was calculated. SD was derived from 20 data points.

### Soft agar colony forming assay

Cells (10^4^) were suspended in culture medium (2 ml) containing 0.3% agar (Difco Bactoagar, Detroit, MI) and plated over a layer of 0.5% agar in the same culture medium in tissue culture plates (35 mm in diameter). Triplicate cultures were incubated at 37°C under 5% CO_2_. Colonies were counted 14 days after plating by viewing under a microscope through a 10× objective and a graduated eyepiece reticle. Any colonies larger than 38 μm in diameter (>10 cells) were scored as positive. SD was derived from three data points.

### Reverse transcriptase–polymerase chain reaction (RT-PCR)

Cerebella were removed from euthanized mice and frozen immediately in liquid nitrogen. Tissue specimens were homogenized in Trizol, and total RNA was extracted using RNeasy Mini Kit (Qiagen, Valencia, CA) according to the manufacturer’s instructions. RT-PCR was carried out using the SuperScript III One-Step RT-PCR kit (Life Technologies, Grand Island, NY) as described previously [25]. In brief, cDNA was synthesized from total RNA by reverse transcription and PCR in the presence of oligonucleotide primers specific for Myc epitope–tagged *Arnt*. RT-PCR products corresponding to the constitutively expressed, glycolytic enzyme glyceraldehyde-3-phosphate dehydrogenase (GAPDH) served as internal controls for sample variation in mRNA degradation and gel loading. Reaction products were separated by electrophoresis through agarose gels and visualized by UV illumination after immersion in ethidium bromide solution.

### Expression profiling and molecular subgrouping of human medulloblastomas

Human primary medulloblastomas were profiled on Affymetrix Genechip Human Exon 1.0ST arrays at The Centre for Applied Genomics in Toronto, Canada (www.tcag.ca). Expression analysis was performed using Affymetrix Expression Console (Version 1.1) as previously described [26]. Additional, publically available medulloblastoma expression data sets were obtained from NCBI Gene Expression Omnibus and used to validate our findings [9,27]. Subgrouping of tumors was performed using an 84-gene expression classifier [28].

### Statistics

We assessed statistical significance of intergroup differences in cell migration, invasion, and anchorage-independent growth using ANOVA and Fisher’s protected least significant difference test. The significance of intergroup differences in the incidence of tumor formation and LMD was assessed using the χ^2^ contingency test.

## Results

### Sleeping Beauty transposon mutagenesis identifies Arnt and Gdi2 as common insertion site genes in metastatic medulloblastomas

**Figure 1:**
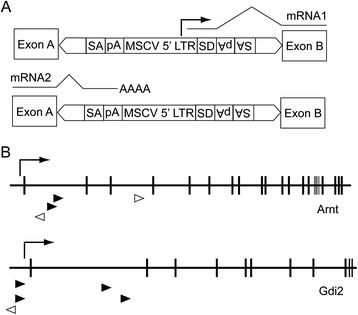
**SB transposon insertions in** ***Arnt*** **and** ***Gdi2*** **.** ***A***, For cancer gene identification, the SB transposon is designed to mimic retroviral insertional mutagenesis. The transposon contains sequences from the 5’ long terminal repeat (LTR) of murine stem cell virus (MSCV) which serve as promoter/enhancer elements to activate transcription of genes into which the transposon has inserted. The MSCV LTR is followed by a splice donor (SD). Thus, a transcript initiated in the LTR (mRNA1) can splice into downstream exons of the target gene. The transposon also contains splice acceptor (SA) sites and polyadenylation (pA) sequences to truncate transcription of target genes (mRNA2). The SB transposon schematized here is the T2/Onc vector [29]. ***B***, Insertion maps showing the sites of transposon insertion (arrowheads) in mouse genes *Arnt* and *Gdi2*. Arrow heads point in the direction of transcription from the MSCV LTR. Transcription-activating insertions in the 5’ untranslated region and intron 1 are indicated by solid arrowheads. Coding exons are represented by vertical lines, introns by the interval spaces. Right-angle arrows indicate the direction of gene transcription.

*Arnt* and *Gdi2* emerged as candidate LMD-inducing genes from a study in which SB transposon mobilization in Math1^+^ GNPs in *Patched*^+/−^ mice caused the typically localized, Shh-driven medulloblastomas to disseminate widely throughout the spinal leptomeninges [19]. The SB transposon (Figure 1A) is designed to promote tumor growth by either enhancing transcription of proto-oncogenes or suppressing transcription of tumor suppressor genes located at insertion sites (reviewed in [29]). Candidate LMD genes were identified by sequencing the insertion sites from spinal metastatic tumors and matched cerebellar tumors and then mapping the sequences back to the mouse genome. This approach revealed 359 gene-centric common insertion sites (gCISs), in which the transposon had inserted more frequently than a calculated background level of random insertion.

To identify the strongest LMD driver genes, we first focused on the 285 gCISs that were present either exclusively in the spinal metastatic tumors or in both the primary tumors and matched metastases. This criterion was used to identify genes whose expression putatively conferred a growth advantage to tumor cells in the microenvironment of the spinal cord. Second, we examined the transposon insertion maps to identify genes whose transcription was most likely stimulated by transposon integration (metastasis-promoting oncogenes). These were genes in which (a) the SB transposon had inserted into the 5’ untranslated region or intron I and (b) the murine stem cell virus promoter of the SB transposon pointed in the direction of gCIS-associated gene transcription. Two such insertions occurred in *Arnt* and four in *Gdi2* (Figure 1B, solid arrowheads).

### Arnt and Gdi2 promote leptomeningeal dissemination in Shh-induced medulloblastomas

**Figure 2:**
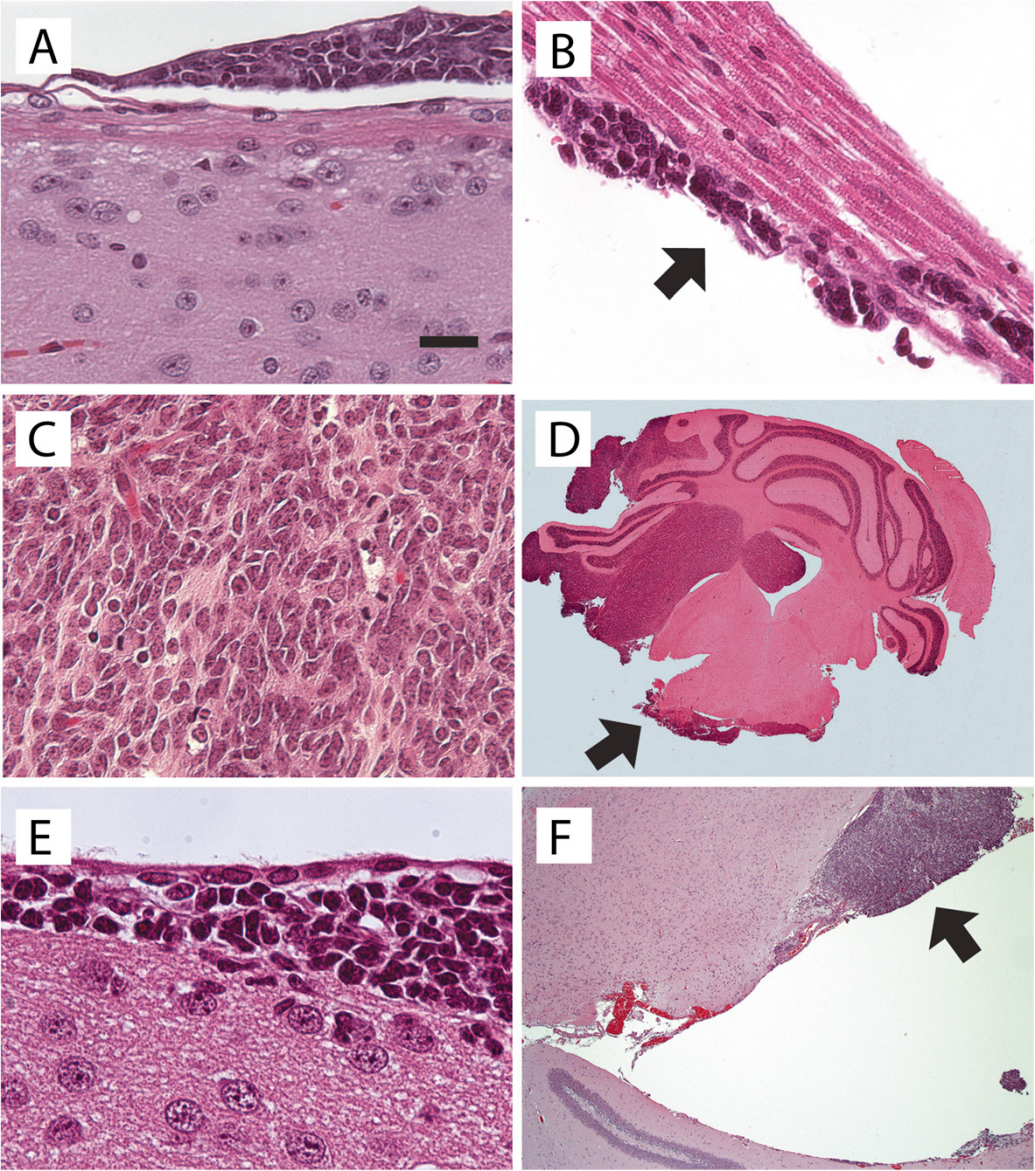
**Histopathology of leptomeningeal dissemination.** ***A***, Aggregates of medulloblastoma cells on the pial surface of the thoracic spinal cord. ***B***, Tumor cells (arrow) attached to the conus medullaris in the lumbar spine. ***C***, Classic medulloblastoma cytoarchitecture showing tumor cells with molded nuclei and mitotic figures. ***D***, Medulloblastoma in the cerebellum spreading into the adjacent fourth ventricle and leptomeninges of the brain stem (arrow). ***E***, Disseminating tumor cells in the subependymal space of the lateral ventricle. Ciliated ependymal cells are visible at the top. ***F***, Nodule of disseminating tumor in the wall of the temporal horn of the lateral ventricle. The dentate gyrus of the hippocampus is visible below. ***A–E,*** *Shh* + *Arnt*–induced tumors. ***F***, *Shh* + *Gdi2*–induced tumors. Scale bar, 25 μm **(** ***A*** ***–*** ***C*** **)**, 16 μm **(** ***E*** **)**, 500 μm **(** ***D*** and ***F*** **)**.

The high frequency of SB transposon insertions in *Arnt* and *Gdi2* indicated that activated transcription of these genes plays a causal role in the LMD program. To test this hypothesis, we used an established mouse model system, in which retroviral transfer of Shh to Nestin^+^ neural progenitor cells in the cerebella of mice induces medulloblastomas that have a very low incidence of metastatic dissemination [18]. Using this system, we transferred and expressed *Arnt* and *Gdi2*, in combination with Shh, in the cerebella of newborn mice and examined histological sections of brain and spinal cord in a 4-month observation period. Our results demonstrated that *Arnt* and *Gdi2* increased the incidence of spinal LMD (Figure 2A,B), as a percentage of mice with histologically verified tumors in the cerebellum, from a baseline of 17% (*Shh* alone) to 67% (*Shh + Arnt*) and 53% (*Shh + Gdi2*) (Table 1). The enhancing effect of *Arnt* on spinal LMD was more potent than we reported previously with *Eras*, *Lhx1*, *Ccrk*, and *Akt*, whereas that of *Gdi2* was comparable [18].

**Table 1:** **Incidence of medulloblastoma formation and leptomeningeal dissemination (LMD) during a 4-month observation period**

**Genes transferred**	**Tumor Incidence**		***P*** value* (spine)
	**Cerebellum**	**Spine LMD†**	
*Shh*	23 of 48 (48%)	4 of 23 (17%)	–
*Shh* + *Arnt*	27 of 69 (39%)	18 of 27 (67%)	0.0005
*Shh* + *Gdi2*	15 of 46 (33%)	8 of 15 (53%)	0.02

*P values were calculated using χ^2^ contingency test to compare spine tumor incidence after combined gene transfer with tumor incidence after transfer of *Shh* alone.

†Spine tumor incidence calculated as a percentage of mice with histologically verified brain tumors.

The incidence of tumor formation in the cerebellum was equivalent in *Shh*, *Shh* + *Arnt*, and *Shh* + *Gdi2* groups (Table 1), indicating that these genes were conferring to tumor cells specific dissemination-enabling traits, rather than simply increasing the number of susceptible cells through enhanced tumor initiation. Addition of *Arnt* and *Gdi2* did not alter the classical cytoarchitecture of Shh-induced medulloblastomas, which is characterized by densely packed sheets of cells with nuclear molding and scant cytoplasm (Figure 2C).

In the clinical setting, spinal LMD is often accompanied by extension of the primary tumor to brain surfaces inside the posterior cranial fossa and to subarachnoid spaces in the forebrain. Accordingly, we observed in mouse medulloblastomas induced by *Shh + Arnt* and *Shh + Gdi2* that tumor cells had spread outside the cerebellum to the leptomeningeal surfaces of the brain stem (Figure 2D) and subependymal spaces of the lateral ventricles (Figure 2E,F).

**Figure 3:**
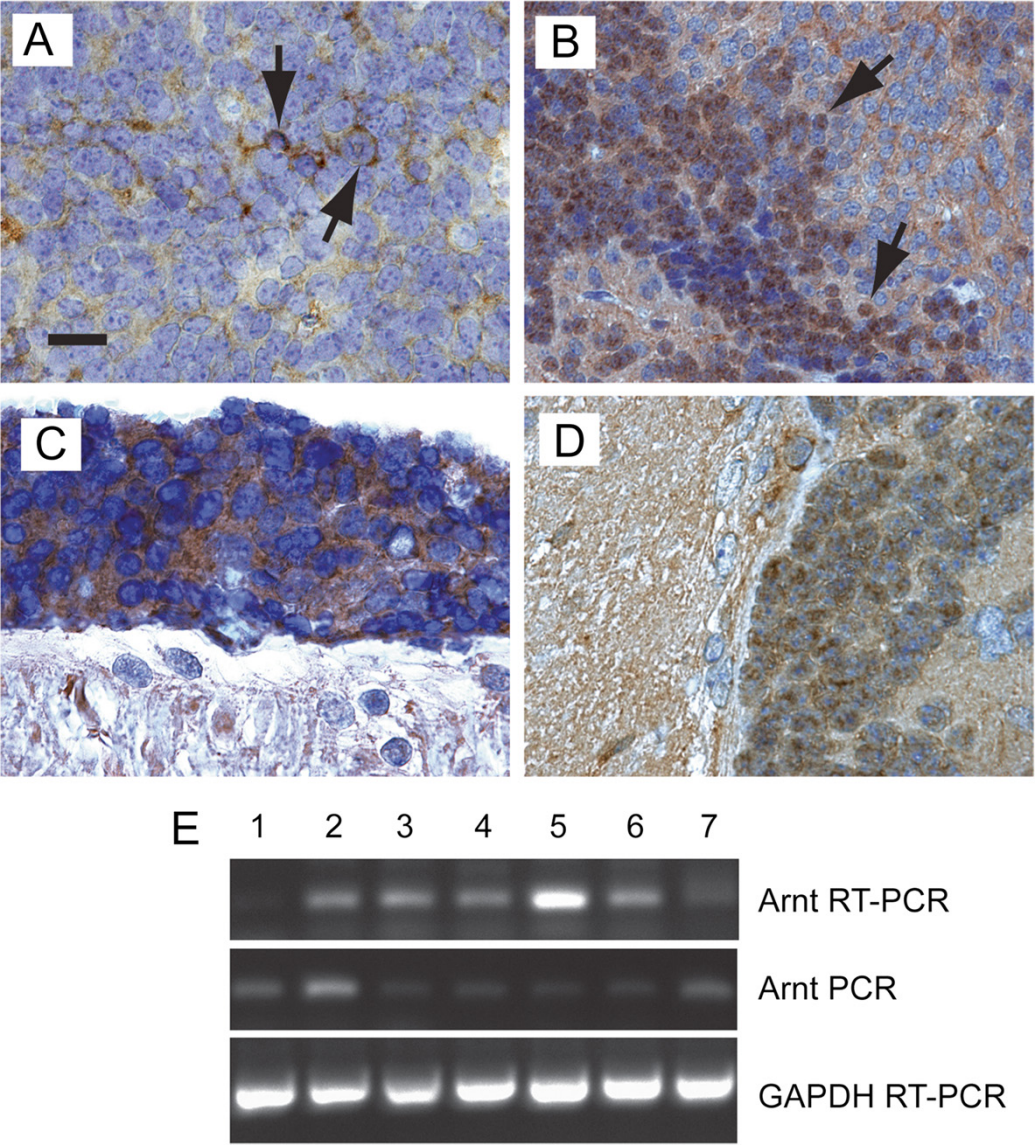
**Expression of RCAS retrovirus–transferred** ***Arnt*** **and** ***Gdi2*** **in tumor cells.** ***A***, Immunoperoxidase staining of GFP in the cytoplasm of *Arnt* + *Gdi2*–induced medulloblastoma cells (arrows) verifying expression of retrovirus-transferred *Gdi2*. ***B***, Immunostaining of medulloblastoma induced by RCAS-mediated transfer of Myc-tagged *Arnt*. Monoclonal antibody 9E10 detects expression of retrovirual Arnt protein in the nuclei of tumor cells (arrows). ***C***, Immunoreactive staining for GFP in the cytoplasm of tumor cells (above) attached to the surface of the spinal cord (below) indicating expression of retrovirus-transferred *Gdi2*. ***D***, Nuclear immunoreactivity for Myc-tagged Arnt in disseminating tumor cells (right) infiltrating the brain stem (left). ***E***, RT-PCR analysis of human *Arnt* sequences in cerebella from mice injected with RCAS-Shh and RCAS-Arnt (*lanes 2–7*) and uninjected mouse (*lane 1*). Reaction products indicate expression of Arnt-Myc fusion protein from the integrated RCAS provirus (*lanes 3–6*). In two cases (*lanes 2 and 7*), band intensity of RT-PCR product was not above the background of a control PCR, indicating that retroviral transfer and integration did not lead to transgene expression in all cases. Scale bar, 16 μm **(** ***A, C–D*** **)**, 50 μm **(** ***B*** **)**.

To verify that the tumor cells expressed the genes transferred by RCAS retrovirus, we used the following approaches. For *Gdi2*, we demonstrated specific immunostaining with an antibody directed against GFP, which was transcribed in tandem with the inserted oncogene through an IRES sequence (Figure 3A). The large size of the *Arnt* coding sequence (2376 base pairs) excluded the possibility of constructing an Arnt-IRES-GFP bicistronic vector. Therefore, we prepared an epitope-tagged version by appending six repeats of a human c-Myc sequence (EQKLISEEDL), which is recognized by monoclonal antibody 9E10, to the carboxy terminus of Arnt, in-frame with the coding sequence. The presence of nuclear immunoreactivity in tumor cells indicated that RCAS-transferred *Arnt* was being expressed and transported to the nucleus, as expected for this transcription factor (Figure 3B). We could not detect RCAS-transferred *Arnt* or *Gdi2* in the spinal metastases by immunostaining in all mice, possibly because individual aggregates of metastasizing cells contained few cells. We could verify that the RCAS-transferred gene was expressed in larger metastases to the spinal cord (Figure 3C) and intracranial sites remote from the cerebellum (Figure 3D).

To provide additional evidence for in vivo expression of retroviral *Arnt*, we performed RT-PCR analysis of cerebellar specimens from six mice that were injected with RCAS vectors carrying Shh and Myc-tagged Arnt and then sacrificed at the onset of neurological signs. We used oligonucleotide primers that spanned a segment of the Arnt–Myc fusion sequence. A reaction product corresponding to Arnt–Myc transgene mRNA was detected in four specimens (Figure 3E). In two specimens, the band intensity of RT-PCR product was not above the background of a control PCR, indicating that retroviral transfer and integration did not lead to transgene expression in all cases.

### Arnt and Gdi2 stimulate motility, invasiveness, and anchorage-independent growth of medulloblastoma cells and tumor precursors

Considering the LMD-promoting effects of *Arnt* and *Gdi2*, we predicted that these genes would confer to tumor cells a set of traits that enable them to detach from the tumor mass, survive a transit phase in the CSF, and ultimately colonize the leptomeninges. Three cell biological traits that are likely prerequisites for tumor cell detachment and CSF dispersal are migration, invasion, and anchorage-independent growth, each of which can be quantified using cell culture assays. Because we wanted to assess the effects of LMD driver genes on these cell traits in Shh-induced medulloblastomas, we tested two cell lines that we considered facsimiles of Shh-driven medulloblastomas and tumor precursors. One was the well-studied, human medulloblastoma line DAOY, which retains a responsive Shh signaling axis [30]. The second was an immortalized, nontransformed cell line derived from mouse GNPs (SHH-NPD cells).

We created the SHH-NPD cell line by first preparing GNP cultures from the cerebella of *Ntv-a* mice on postnatal day six, using an established protocol that generates cultures that are 90% enriched for GNPs [31]. GNPs can survive in culture for two weeks, but only when exogenous Shh is added to the medium [32]. We then infected the Nestin^+^ cells in this culture with an RCAS retrovirus to express Shh constitutively. SHH-NPD cells proliferate continuously in serum-containing medium, but they do not form tumors after subcutaneous injection in athymic mice or intracerebral implantation in immunocompetent mice. Reflecting their origin from GNPs, SHH-NPD cells express βIII-tubulin, a marker of early neuronal differentiation, but not neurofilament protein or glial fibrillary acidic protein, markers of terminally differentiated neurons and astrocytes, respectively (Additional file 1: Figure S1).

**Figure 4:**
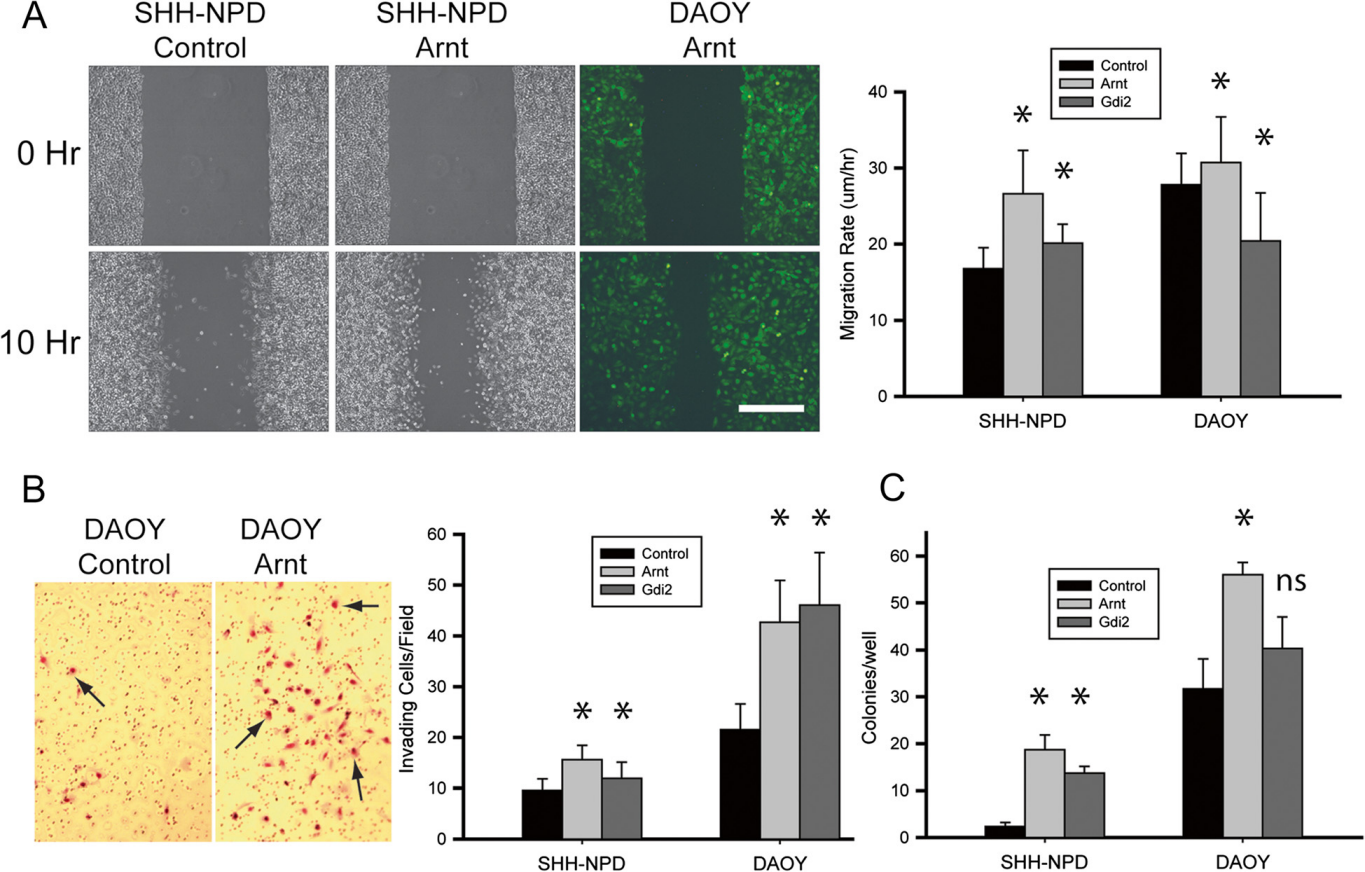
**Analysis of migration, invasion, and anchorage-independent growth in DAOY and SHH-NPD cells.** ***A***, *In vitro scratch assay* in which monolayers of SHH-NPD and DAOY cells, previously infected with HIVZsGreen lentivirus for stable expression of *Arnt*, were scratched to create a linear wound and photographed 0 and 10 hours later. The average rate of cell migration (μm/hr) across the wound gap was determined by measuring the distance between opposing cell fronts. Fluorescence microscopy verified expression of ZsGreen-tagged *Arnt* and Gdi2 in the migrating cells. Bar graphs show the mean rate of cell migration (μm/hr) across the gap. ***B***, *Matrigel chemoinvasion assay* in which SHH-NPD and DAOY cells expressing lentivirus-transferred *Arnt* and *Gdi2* or empty virus (control) were placed in the upper wells of BD Biocoat Matrigel chambers and the cells invading through the matrigel to the lower chamber (arrows) were counted. Bar graphs show the mean number of invading cells/microscope field in two separate experiments. ***C***, *Soft agar colony forming assay*. Bar graphs show the mean number of colonies (>38 μm) per well (35-mm diameter) 14 days after plating in 0.3% agar. Error bars, SD. Scale bar, 400 μm. Statistical significance, compared with control, indicated by asterisks (*P* < 0.05) or “ns” (not significant).

To assess cell migration, we used the in vitro scratch assay, which measures the rate of cell movement across a gap created in a confluent monolayer of cells growing on the surface of a tissue culture plate [33]. We overexpressed *Arnt* and *Gdi2* in DAOY and SHH-NPD cells using the HIV-ZsGreen lentivirus expression system [24]. The average rate of cell migration across the scratch wound was calculated and compared with that of control cells infected with empty lentivirus (Figure 4A). The mean migration rate of control DAOY cells (24.6 μm/hr) was greater than that of SHH-NPD cells (16.8 μm/hr), consistent with the idea that increased motility is a general feature of transformed cells. Addition of Arnt increased the migration rate of DAOY and SHH-NPD cells 1.2-fold and 1.6-fold, respectively (*P* < 0.0001 by ANOVA for both cell lines). Gdi2 increased the migration of SHH-NPD cells 1.2-fold and paradoxically decreased that of DAOY cells (*P* < 0.0001 by ANOVA for both cell lines).

To measure invasiveness, we used the matrigel chemoinvasion assay, in which the percentage of cells invading through a gel matrix is determined as a function of time. Because matrigel contains the principal macromolecular components of epithelial basement membranes, the ability of cells to invade through this barrier has long been considered a reliable experimental model of the initial step in metastasis—transgression of the basement membrane [34]. Results of the chemoinvasion assays showed that expression of *Arnt* and *Gdi2* enhanced the ability of both SHH-NPD and DAOY cells to penetrate the matrigel barrier (Figure 4B). For example, *Arnt* increased invasiveness of SHH-NPD cells 1.6-fold and DAOY cells two-fold (*P* < 0.0001 by ANOVA).

A general feature of transformed cells is the ability to proliferate without attachment to a solid surface (anchorage-independent growth). This would seem to be an essential attribute of disseminating medulloblastoma cells, which must survive passage through the CSF before implanting on the pial surfaces of the spinal cord. To assess anchorage-independent growth, we measured the efficiency with which DAOY and SHH-NPD cells formed colonies in soft agar before and after lentivirus-mediated expression of *Arnt* and *Gdi2* (Figure 4C). The ability of SHH-NPD cells to proliferate in soft agar was very low (2 colonies/well), as expected for a nontransformed cell, whereas expression of *Arnt* and *Gdi2* increased colony forming efficiency 8-fold and 6-fold, respectively. In the transformed DAOY cell line, *Arnt* increased colony formation from a baseline of 32 to 56 cells/well (*P* = 0.02), whereas *Gdi2* had no significant effect. The ability of *Arnt* and *Gdi2* to promote anchorage-independent growth is not derived from a general mitogenic effect because neither gene stimulated the proliferation of adherent cells when tested in an MTT colorimetry assay (data not shown).

### Expression of *ARNT* and *GDI2* in human medulloblastomas

We asked whether increased expression of *ARNT* and *GDI2* was associated with an increased risk of metastasis in medulloblastoma patients. To answer this question, we analyzed two publicly accessible sets of gene expression data on human medulloblastomas, which had been assigned to one of four molecular subgroups (WNT, SHH, Group 3, and Group 4). Previously published work showed that metastasis (defined by the presence of microscopic tumor cells in the CSF, radiographically detected LMD, or metastasis outside of the central nervous system) was significantly more common in Group 3 (46.5%) and Group 4 (29.7%) than in the WNT (17.9%) and SHH (19.1%) subgroups [28]. The patient tumor specimens had been collected through two multicenter consortia, the Medulloblastoma Advanced Genomics International Consortium (MAGIC) [35] and the Children’s Oncology Group (COG) [7]. Metastasis stage (M-stage) was recorded for 285 patients in the MAGIC cohort and 189 in COG, using the five-tiered Chang classification, based on CSF cytology and magnetic resonance imaging [36].

**Figure 5:**
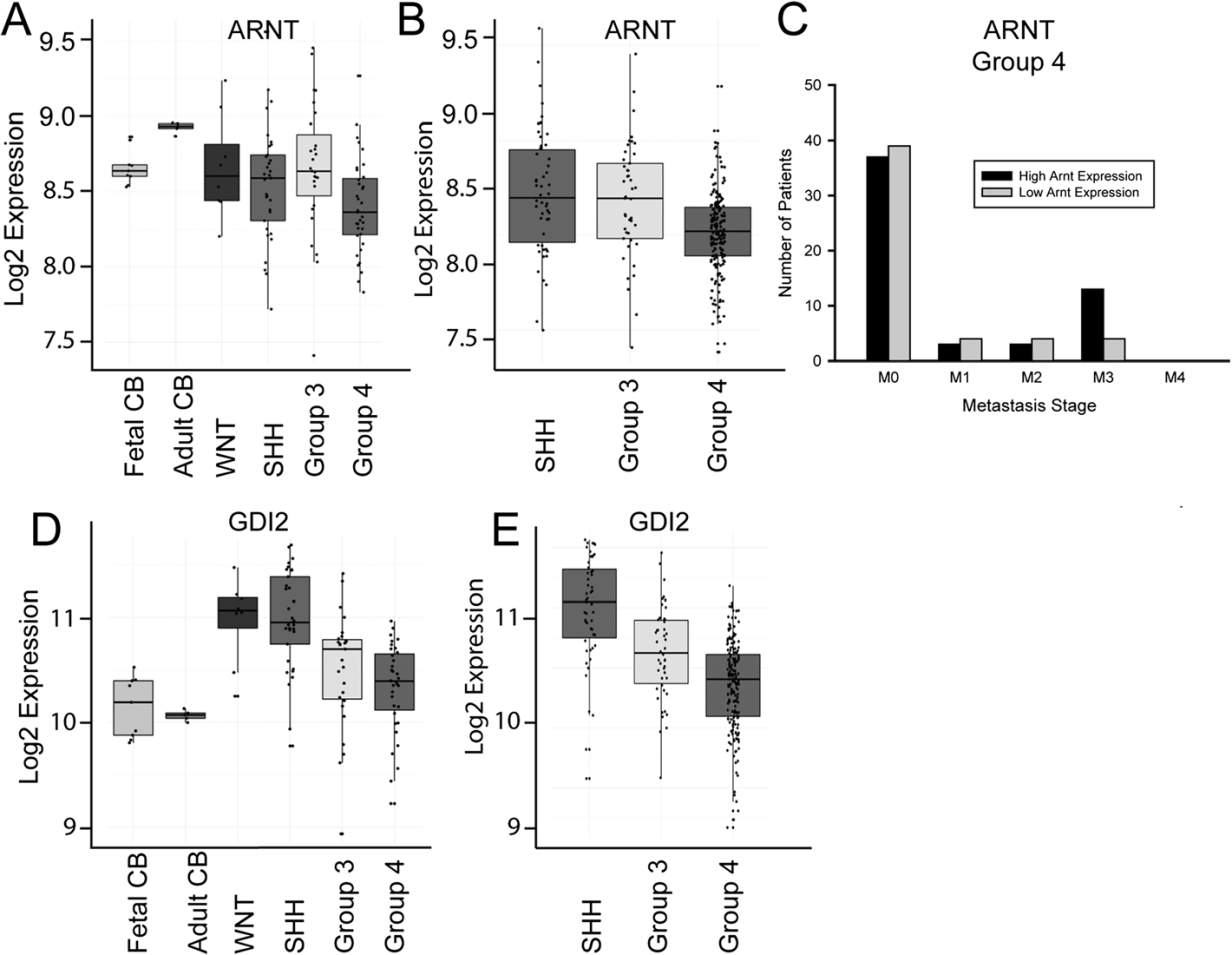
**Expression of** ***ARNT*** **and** ***GDI2*** **in human medulloblastoma subgroups.** Box plots showing relative expression of *ARNT* **(** ***A*** **,** ***B*** **)** and *GDI2* **(** ***D*** **,** ***E*** **)** in normal cerebella (CB; fetal n = 9, adult n = 5) and medulloblastoma samples from the MAGIC consortium profiled on Affymetrix exon arrays. The 103 medulloblastomas in the discovery set **(** ***A*** ***,*** ***D*** **)** and the 285 in a nonoverlapping validation set **(** ***B*** ***,*** ***E*** **)** were classified according to molecular subtype (WNT, SHH, Group 3, Group 4). Log2 expression is a measure of the luminosity of the gene probe signal, corrected for the background luminosity of each array and normalized using control probes across different arrays. ***C***, Bar graph correlating M-stage with *ARNT* mRNA level (high = top quartile; low = bottom quartile) in Group 4 medulloblastomas (n = 200).

We determined the relative levels of *ARNT* mRNA in two sets of patients from the MAGIC cohort: a 103-patient discovery set, which was divided into four molecular subgroups (Figure 5A), and a nonoverlapping 285-patient validation set, which was divided into three non-WNT subgroups (Figure 5B). An identical comparison was made among the four tumor subgroups in COG (Additional file 2: Figure S2). The mean level of *ARNT* expression in the tumors was comparable with that of normal fetal cerebellum. Approximately 5% of medulloblastomas analyzed in a subgroup–dependent or –independent fashion showed >1.5-fold increased expression relative to normal fetal cerebellum.

When we correlated M-stage with *ARNT* expression levels in tumor subgroups from the combined MAGIC and COG series, we found that among 20 patients with Group 4 tumors, there were more cases of spinal LMD (M3-stage) in tumors showing high *ARNT* expression (top quartile) than low expression (bottom quartile) (χ^2^*P* = 0.02) (Figure 5C). We did not see an association between ARNT expression levels and M-stage for SHH and Group 3 tumors.

Comparative analysis of *GDI2* expression in the human tumor data sets revealed that *GDI2* mRNA levels were higher in medulloblastomas than in normal adult and fetal cerebellum (MAGIC discovery and validation data shown in Figure 5D and E, respectively, and COG data in Additional file 2: Figure S2). Subgroup analysis showed that *GDI2* expression was highest in SHH tumors, consistent with our experimental results in Shh-induced tumors in mice. In contrast to *ARNT*, there was no correlation between *GDI2* expression and M-stage across any of the molecular subgroups.

An unanswered question is whether expression of LMD-driving genes increases over time in a subpopulation of tumor cells, leading to metastatic dissemination at a later time point. Answering this question will require analyzing medulloblastoma cells that have already disseminated, a project that is hindered by the fact that surgical excision of metastatic tumors is rarely indicated in patients. We profiled matched pairs of primary and metastatic medulloblastomas from eight patients on Affymetrix exon arrays (Additional file 3: Figure S3). *ARNT* mRNA levels were increased ≥1.2-fold in metastatic compared with primary tumors in four patients (50%), and *GDI2* in six (75%). Although this observation is consistent with a selective growth advantage conferred to cells by *ARNT* and *GDI2*, larger sets of primary-metastatic tumor pairs will be needed to verify this trend.

## Discussion

We show here that ectopic expression of *Arnt* and *Gdi2*, which were identified in an unbiased genetic screen for metastasis genes using SB transposon mutagenesis, promotes LMD in Shh-induced medulloblastomas in mice. When overexpressed in cell culture models of Shh-driven medulloblastomas and tumor precursors, *Arnt* stimulated motility, invasiveness, and anchorage-independent growth, cell traits that are closely associated with metastatic competence in carcinomas. *Gdi2* had these same effects on tumor precursors and simulated invasiveness in fully transformed medulloblastoma cells.

Although Arnt and Gdi2 have previously been implicated in cancer biology, the molecular mechanisms by which these proteins cause medulloblastoma cells to disseminate remain uncertain. Arnt is most widely known in oncology as the constitutively expressed binding partner of the hypoxia-inducible transcription factors HIF1α and HIF2α, whose downstream target genes play active roles in angiogenesis, proliferation, invasion, and metastasis (reviewed in [37]). Arnt occupies a second sphere of biology through its interaction with the aryl hydrocarbon receptor (AhR), which binds dioxin and other xenobiotics. After binding a xenobiotic molecule, AhR translocates from its normal location in the cytoplasm to the nucleus, where it dimerizes with Arnt and activates transcription of genes containing dioxin-response elements.

A substantial body of evidence indicates that the role of Arnt/AhR signaling in cell biology is not restricted to the xenobiotic response but rather extends to normal developmental processes and tumorigenesis (reviewed in [38]). During mouse cerebellar development, for example, both Arnt and AhR are highly expressed in GNPs during their active proliferation phase on postnatal days 5–6, suggesting that Arnt might maintain GNPs and possibly derivative medulloblastoma cells in an undifferentiated state [39]. We do not know whether the LMD-inducing effects of Arnt are mediated by HIF or AhR signaling. Arguing against HIF as the relevant pathway, we could not detect increased expression of two known HIF transcription targets, vascular endothelial growth factor and glucose transporter-1, by immunostaining sections of Shh + Arnt–induced medulloblastomas (data not shown).

Gdi2 prevents the dissociation of GDP from Rab proteins, a family of membrane-localized GTPases, which switch between active GTP-bound and inactive GDP-bound states. There is extensive literature to support the general concept that Rab proteins coordinate the intracellular movement of membrane vesicles (reviewed in [40]). The function of individual Rab family members is specified by their subcellular localization. By inhibiting GDP release, Gdi2 maintains Rab in an inactive conformation, an action that seems uncharacteristic for an oncoprotein. Nevertheless, Gdi2 has a second, more oncogenic role as a chaperone protein, facilitating the transport of Rab molecules from inactive reserve stores in the cytosol to active sites in their specific membrane compartments [41,42]. Certainly, Rab GTPases contribute directly to cancer cell physiology. Rab25, for example, promotes invasive, metastasis-like migration of cells by binding α5β1 integrin molecules and directing their localization to the tips of extending pseudopods [43].

Extrapolation of our cell culture results to the in vivo growth of medulloblastoma supports a mechanistic scheme whereby *Arnt* and *Gdi2* cause shedding of cells from the primary tumor mass into the CSF by increasing cell motility and invasiveness. By making tumor cells competent to proliferate without surface attachment, expression of *Arnt* and *Gdi2* would also favor the formation of suspended colonies of cells capable of surviving in the CSF before reimplanting on a leptomeningeal surface. The ability of medulloblastoma cells to circumvent apoptotic programs normally triggered by detachment from a solid surface (anoikis) is most likely a prerequisite for LMD. This adaptive response is analogous to that of metastasizing carcinoma cells, which must survive the passage through the bloodstream before colonizing a distant organ.

We could not determine in all cases whether the presence of tumor cells in the brain stem and forebrain resulted from direct extension from the primary tumor or dispersal through the CSF. Nevertheless, the cell traits that empower cells to disseminate to the spinal column (motility, invasiveness, and anchorage-independent growth) could also promote extension from the cerebellum to the fourth ventricle or remote sites in the forebrain.

We demonstrated the LMD-inducing effects of *Arnt* and *Gdi2* in Shh-induced medulloblastomas in vivo and validated these effects in culture using cell lines in which the Shh signaling pathway is active. Therefore, we cannot conclude that *Arnt* and *Gdi2* are LMD drivers in Shh-independent medulloblastomas even though *Arnt* and *Gdi2* are expressed in all subgroups. Nevertheless, the fact that medulloblastomas of any molecular subgroup can metastasize, either at initial presentation or relapse, broadens the clinical significance of LMD driver genes discovered in different genetic backgrounds [44].

The multiple cellular transitions connecting tumor stromal detachment, CSF passage, and finally leptomeningeal spreading indicate that LMD might approach the complexity of the invasion–metastasis model used to describe hematogenous metastasis of epithelial cancers. Transposon mutagenesis revealed hundreds of candidate genes, although we do not know how many of these genes are drivers as opposed to passengers in the LMD process. Through the functional validation studies we have reported here and previously [18], we know that Shh-induced medulloblastomas can start down a path of disseminated growth by addition of single LMD driver genes.

## Additional files

Additional file 1: Figure S1. Characterization of SHH-NPD cells. ***A***, Immunoperoxidase staining showing expression of c-Myc–tagged Shh (Mab 9E10). ***B***, Western blot analysis of SHH-NPD cells. Two bands (60 and 37 kDa), corresponding to full-length Shh and carboxy-terminal peptides derived from autoproteolysis [45], were detected with an antibody directed against the c-Myc epitope tag of RCAS-Shh in SHH-NPD cells (*lane 2*), but not in uninfected GNPs (*lane 1*). ***C***, Expression of βIII-tubulin, a marker of early neuronal differentiation. ***D***, Absence of immunoreactive staining for neurofilament protein, a marker of post-mitotic neurons. Scale bar, 16 μm. Additional file 2: Figure S2. Expression of *ARNT* and *GDI2* in human medulloblastoma subgroups. Box plots showing relative expression of *ARNT* **(** ***A*** **)** and *GDI2* **(** ***B*** **)** in normal adult cerebella (CB; n = 10) and medulloblastoma samples (n = 187) from the COG consortium profiled on Affymetrix exon arrays and classified according to molecular subtype (WNT, SHH, Group 3, Group 4). Additional file 3: Figure S3 Differential expression of *ARNT* and *GDI2* in matched pairs of primary and metastatic tumors from eight medulloblastoma patients.
