# Supplementary figures and images for: Genetic drivers of metastatic dissemination in sonic hedgehog medulloblastoma
Source: Acta Neuropathol Commun. 2014 Jul 25;2:85. doi: 10.1186/s40478-014-0085-y (PMC4149244; doi:10.1186/s40478-014-0085-y)

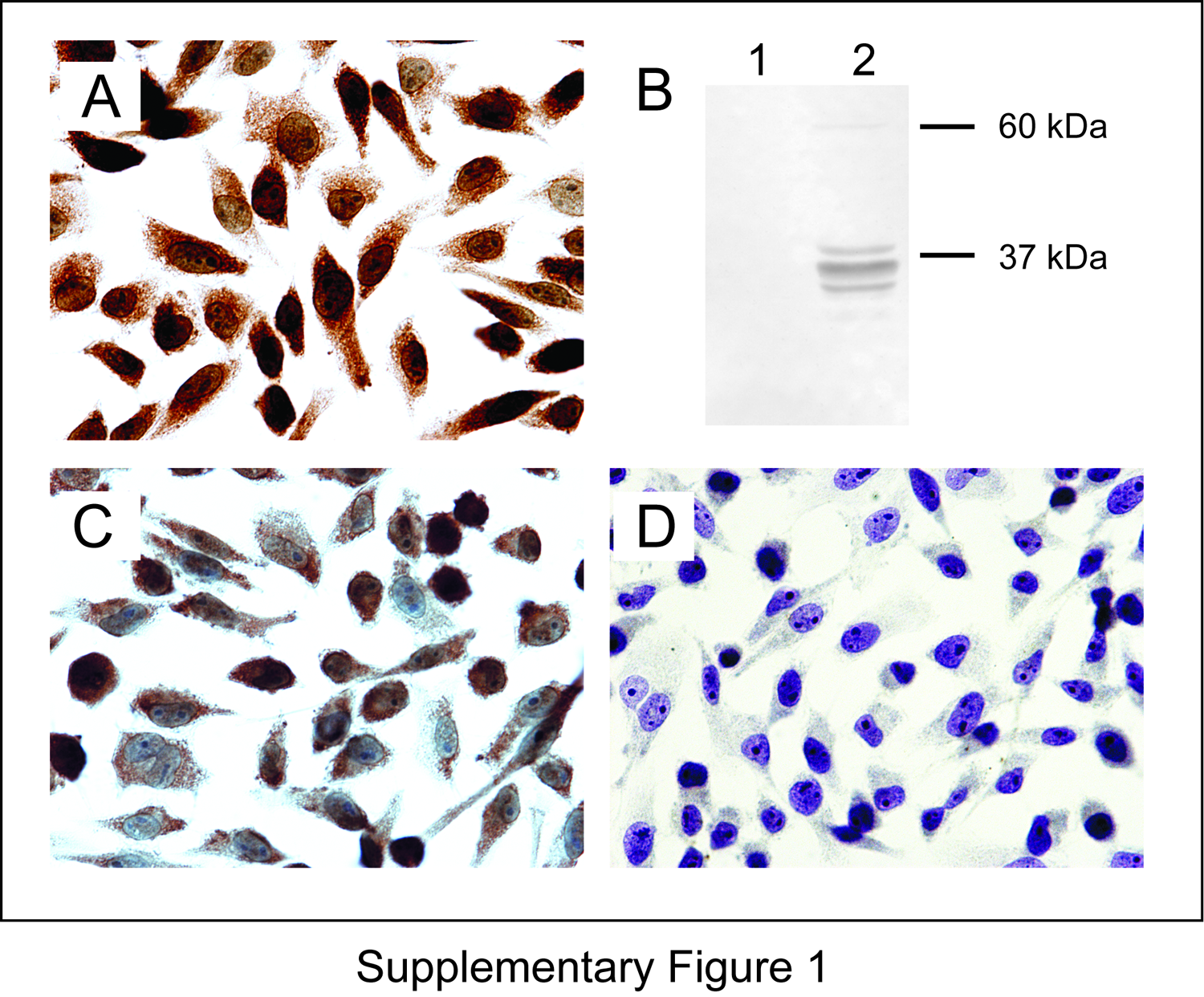

Supplement: Additional file 1: Figure S1. — Characterization of SHH-NPD cells. A, Immunoperoxidase staining showing expression of c-Myc–tagged Shh (Mab 9E10). B, Western blot analysis of SHH-NPD cells. Two bands (60 and 37 kDa), corresponding to full-length Shh and carboxy-terminal peptides derived from autoproteolysis [45], were detected with an antibody directed against the c-Myc epitope tag of RCAS-Shh in SHH-NPD cells (lane 2), but not in uninfected GNPs (lane 1). C, Expression of βIII-tubulin, a marker of early neuronal differentiation. D, Absence of immunoreactive staining for neurofilament protein, a marker of post-mitotic neurons. Scale bar, 16 μm. [file 40478_2014_85_MOESM1_ESM.tiff]

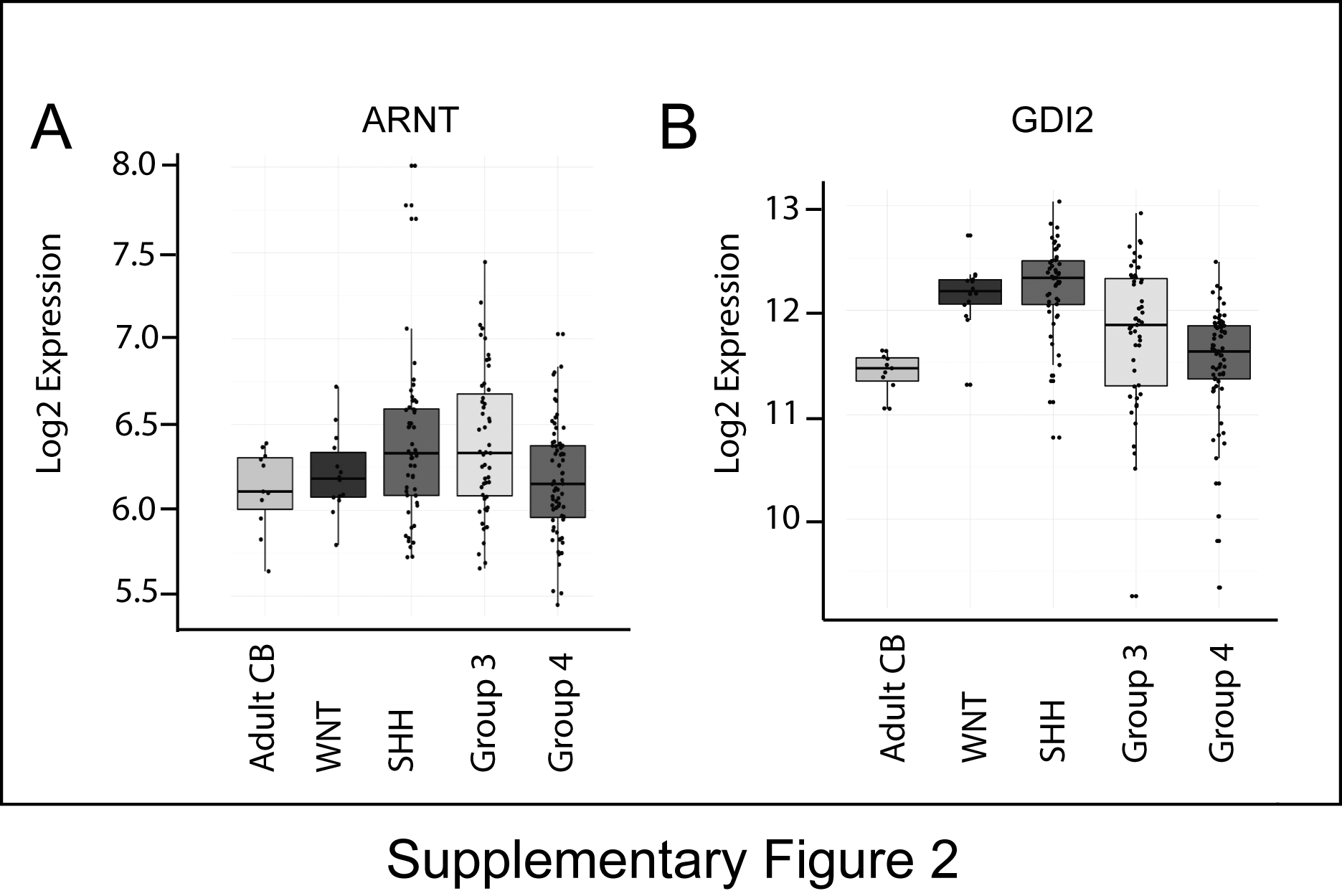

Supplement: Additional file 2: Figure S2. — Expression of ARNT and GDI2 in human medulloblastoma subgroups. Box plots showing relative expression of ARNT ( A ) and GDI2 ( B ) in normal adult cerebella (CB; n = 10) and medulloblastoma samples (n = 187) from the COG consortium profiled on Affymetrix exon arrays and classified according to molecular subtype (WNT, SHH, Group 3, Group 4). [file 40478_2014_85_MOESM2_ESM.tiff]

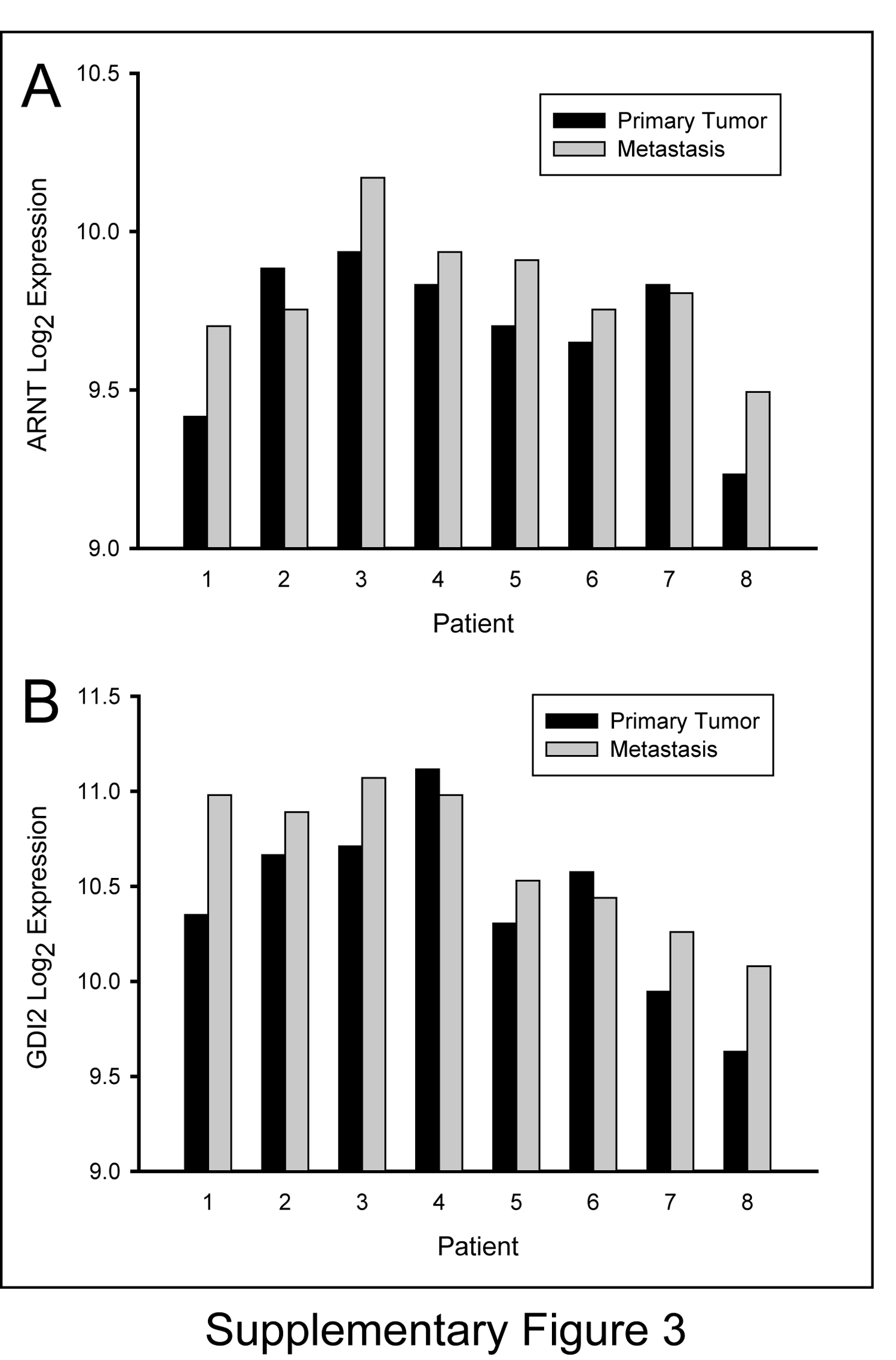

Supplement: Additional file 3: Figure S3 — Differential expression of ARNT and GDI2 in matched pairs of primary and metastatic tumors from eight medulloblastoma patients. [file 40478_2014_85_MOESM3_ESM.tiff]
